# Endoscopic Procedures in the Treatment of Ureteroenteric Anastomotic Strictures: A Systematic Review and Meta-Analysis

**DOI:** 10.3389/fsurg.2021.626939

**Published:** 2021-04-14

**Authors:** Xun Lu, Yiduo Wang, Qi Chen, Di Xia, Hanyu Zhang, Ming Chen

**Affiliations:** ^1^Department of Urology, Affiliated Zhongda Hospital of Southeast University, Nanjing, China; ^2^Department of Interventional Radiology, Affiliated Zhongda Hospital of Southeast University, Nanjing, China; ^3^Reproductive Medicine Center, Affiliated Zhongda Hospital of Southeast University, Nanjing, China

**Keywords:** ureteroenteric anastomotic strictures, endoscopic procedure, meta-analysis, success rate, systematic review, complications

## Abstract

**Objective:** To evaluate the safety and efficacy of endoscopic procedures for ureteroenteric anastomotic strictures (UESs) after radical cystectomy and urinary diversion.

**Methods:** We performed a meta-analysis of relevant articles through March 2020 using PubMed, Embase, and Cochrane Central Register to assess the efficacy of endoscopic procedures in UES according to the PRISMA and PICOS criteria. The main endpoints were success rate and complications, and we also compared the efficacy of different methods and stricture length and side in UES. Cochrane Collaboration's Revman version 5.3 and Stata version 15.1 software were used for statistical analysis.

**Results:** A total of 18 retrospective studies with 697 patients were included. The median follow-up ranges from 12 to 62.5 months. Patients treated with endoscopic procedures had an overall success rate of 46%. The pooled rate of Clavien–Dindo ≥ 3 complications was 3.8% among included studies. Laser vaporization and stent insertion (48 and 47%) had a relatively high success rate than balloon dilatation (35%). In subgroup analysis, the success rate of endoscopic procedures for ≤ 1-cm strictures was significantly higher than that for >1-cm ones [odds ratio (OR), 8.65; 95% confidence interval (CI), 3.53–21.21; *P* < 0.00001]. In addition, the success rate in cases with strictures of the right side was relatively higher than that in cases with strictures of the left side (OR, 1.72; 95% CI, 1.05–2.81; *P* = 0.03).

**Conclusion:** Our pooled studies showed that endoscopic operation is feasible and associated with a moderate success rate along with a relatively low incidence of perioperative complications in the treatment of UES, especially with length ≤ 1 cm and right side. Although there is still no consensus on endoscopic technique for UES regarding balloon dilatation, stent insertion, and laser vaporization, we believe that endoscopic management is a safety and available approach for UES with close follow-up.

## Introduction

For muscle-invasive and high-risk non-muscle-invasive bladder cancer, radical cystectomy, and urinary diversion are recommended (1). Considering quality of life, most of the bladder cancer patients choose ileal conduit or orthotopic neobladder for substitution. However, regardless of the method of diversion, all involve the anastomosis of the ureters to bowel.

It has been reported that the incidence of strictures after ureteroenteric anastomotic is about 10% (2), of which the majority are benign. The ureteroenteric anastomotic stricture (UES) is probably caused by periureteral fibrosis or scarring secondary to ischemia (3–5). Most of the strictures occur with median time to diagnosis in the literature reported as 7–25 months after surgery; such a time interval indicates that it is a long-term complication after urinary diversion (6–8). The severe consequences of strictures are infection, reduced glomerular filtration rate, and even loss of renal function (9). Given such serious consequences, it is essential for the treatment of strictures.

With the advances in endourology, the management of UES renders urologists multiple alternatives such as holmium (Ho):YAG laser vaporization, endoureterotomy, stent insertion, and balloon dilatation. Kramolowsky et al. (10) had first reported the outcomes between balloon dilatation and open repair in the management of UES. While endoscopic procedures for UES had a relative low success rate than open revision in follow-up, the decreased operation time, complications, and shorter hospitalization time associated with the endourological approach favor its use over open revision. Series of studies reporting the outcomes of endoscopic procedures in UES have been published; therefore, we aimed to synthesize the evidence-based data to assess the therapeutic effect of endoscopic procedures in UES by performing a systematic review and meta-analysis.

## Methods

### Literature Search

According to the PICOS criteria (P: patients with UES; I: endoscopic management; C: different methods of endoscopic approach; O: success rate and major complication rate; S: retrospective analysis), we performed subject terms (MeSH) including “ureteroenteric anastomotic strictures” with their single words to search for relevant articles through March 2020 in PubMed, Embase, and Cochrane Central Register. The complete search used for PubMed was (“ureteroenteric anastomotic stricture” OR “ureteroenteric anastomotic stenosis” OR “ureteral obstruction”) AND (“endoscopic procedures” OR “balloon dilatation” OR “laser vaporization” OR “endoureterotomy”). We also manually probed the references of included studies to recognize potential ones. Two authors independently searched and screened articles. Our search strategy followed by PRISMA flow diagram was shown in Supplementary Table 1. If any disagreement exists, we will make a discussion to reach a consensus.

### Data Extraction

The inclusion criteria were as follows: retrospective/prospective/RCT trails, English language, full-text articles, and studies that evaluated overall success rate of endoscopic procedures in the treatment of UES. The diagnosis of UES was confirmed by imaging or ureteroscopy. Case reports, reviews, abstracts, animal experiments, and letters were excluded. One reviewer extracted the study authors, date of publication, level of evidence, surgical method, number of patients treated with endoscopic procedures, stricture length and side, complications, and overall success rate. Data were then verified by another reviewer. The definition of overall success was no radiographic sign of obstruction of the upper urinary tract, absence of infection or flank pain, and no need for nephrostomy tube placement during follow-up.

### Quality Assessment and Statistical Analysis

In preparing this review and meta-analysis, we strictly followed the PRISMA and PICOS criteria (Supplementary Table 2). We used methodological index for non-randomized studies (MINORS) to assess the methodological quality of included non-randomized studies (11). Our meta-analysis was performed through Stata software, version 15.1 (StataCorp, College Station, TX, USA) and Review Manager software, version 5.3 (Cochrane Collaboration, Oxford, United Kingdom). First, heterogeneity between studies was assessed using the *Q* statistic. If there was no significant heterogeneity (*P* ≥ 0.1, *I*^2^ ≤ 50%), the fixed effect model was used for pooled analysis. If heterogeneity existed (*P* < 0.1, *I*^2^ > 50%), we first tried to identify the source of heterogeneity and conducted subgroup pooled analysis. If the heterogeneity could not be eliminated, the random effect model was used for pooled analysis. We combined outcome measuring overall success rate and complications of surgery, reported as mean ± standard error (SE). Subgroup analyses were performed to compare the influence of stricture length (≤ 1 vs. >1 cm) and stricture side (right vs. left) on success rate using the pooled odds ratio (OR) and confidence interval (CI) as risk estimates.

## Results

### Study Characteristic

A total of 18 studies with 697 patients were finally included in this meta-analysis (4, 12–28). All studies included were retrospective designed. The median follow-up ranges from 12 to 62.5 months. The MINORS index was assessed for all included studies, which showed relatively high or medium quality. Eight articles reported balloon dilation, and seven articles reported laser vaporization, while stent insertion alone was used in six studies. We summarized our results in Table 1.

**Table 1:** Characteristics of included studies.

**References**	**Design**	**Surgical method**	**No. of patients**	**Success rate**	**Length**		**Side**		**Complication ≥3**	**Follow-up (median)**	**MINORS**
					**≤1 cm**	**>1 cm**	**Left**	**Right**			
van Son et al. (12)	Retrospective	B/L/S	135	38.90%	NA	NA	NA	NA	4	34	10
Ahmed et al. (13)	Retrospective	S	29	44.83%	NA	NA	NA	NA	NA	23	11
Gomez (14)	Retrospective	B/L/S	28	71.40%	7	21	NA	NA	5	25	12
Campschroer et al. (15)	Retrospective	S	56	41.07%	NA	NA	34	22	NA	37.7^*^	11
Schondorf (16)	Retrospective	B/L/S	96	26.04%	44	52	64	32	9	29	10
Nassar and Alsafa (4)	Retrospective	S	37	51.35%	9	8	27	10	NA	47	12
Milhoua et al. (17)	Retrospective	B/L	21	27.27%	9	10	15	5	NA	21	12
Tal et al. (18)	Retrospective	S	28	45.00%	NA	NA	NA	NA	1	62.5	12
Laven et al. (19)	Retrospective	L	16	50.00%	NA	NA	9	7	NA	35	11
Poulakis et al. (20)	Retrospective	E	43	60.47%	NA	NA	27	16	NA	38.8^*^	12
Watterson et al. (21)	Retrospective	L	24	70.83%	NA	NA	NA	NA	NA	22	10
Dimarco et al. (22)	Retrospective	B	52	15.00%	22	16	34	15	1	24^*^	11
Laven et al. (23)	Retrospective	L	19	57.00%	NA	NA	7	9	NA	20.5	12
Lin et al. (24)	Retrospective	B	10	30.00%	6	4	9	1	NA	24	10
Ravery et al. (25)	Retrospective	B	14	61.54%	NA	NA	NA	NA	NA	15	10
Cornud et al. (26)	Retrospective	E	37	71.00%	NA	NA	NA	NA	NA	12	12
Meretyk et al. (27)	Retrospective	E	14	57.14%	4	10	NA	NA	1	28.6^*^	10
Shapiro et al. (28)	Retrospective	B	37	16.22%	NA	NA	NA	NA	NA	12	11

*B, balloon dilation; L, laser vaporization; S, stent insertion; E, endoureterotomy; NA, not applicable; MINORS, methodological index for non-randomized studies*.

* *Mean follow-up*.

### Overall Success Rate

**Figure 1:**
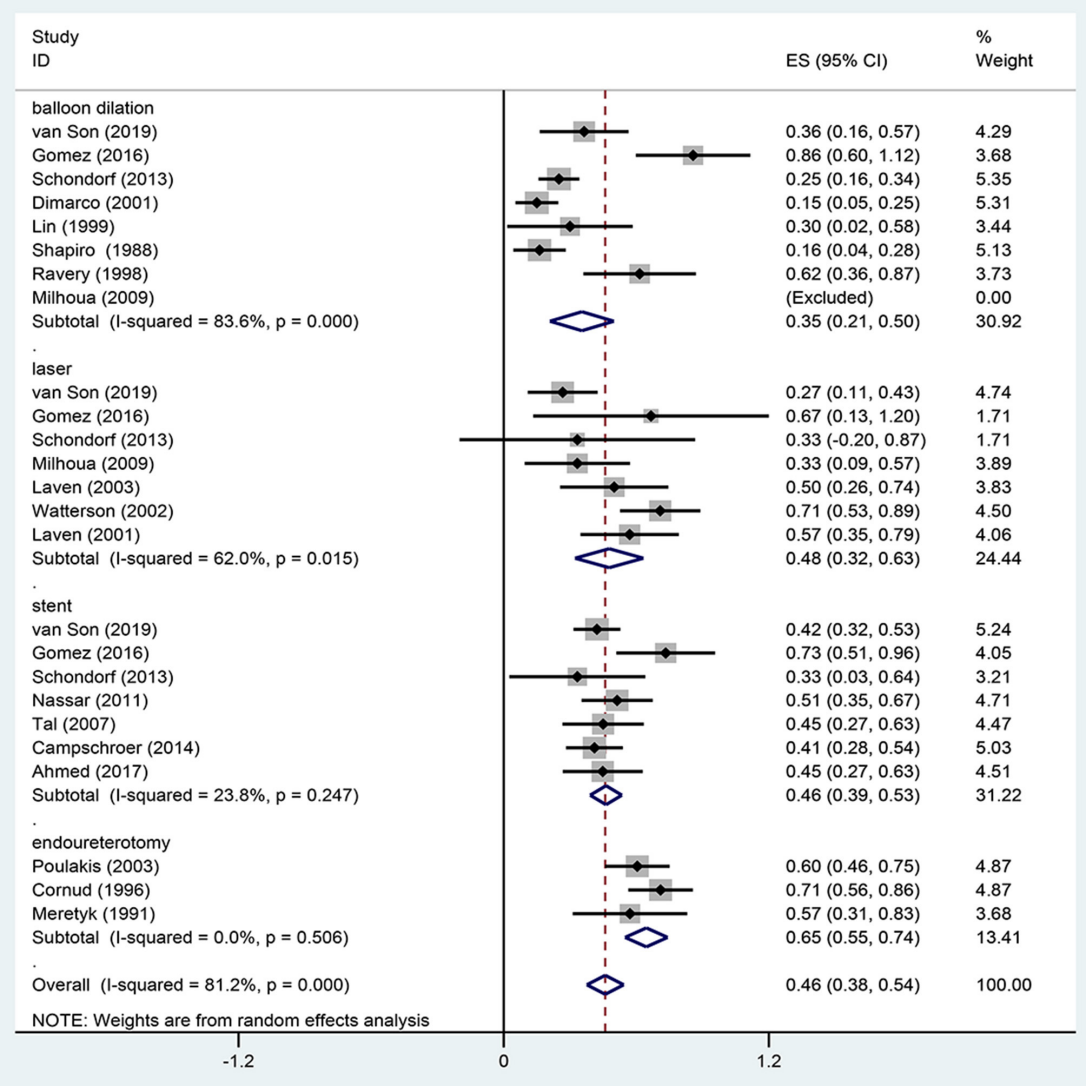
Overall success rate of included studies.

Eighteen studies reported the overall success rate of endoscopic management in UES, of which five were published before 2000. Meta-analysis using a random-effects model showed a technical success rate of 46 ± 9%. Our pooled estimate showed significant heterogeneity (*I*^2^ = 89%), which may come from different surgery methods among studies. Therefore, we performed a subgroup analysis. In subgroup analysis according to different surgical methods of UES, the average success rate of balloon dilatation was 35%, which had a relative low success rate compared to other methods. However, laser vaporization and stent insertion had similar results (48 vs. 47%) (Figure 1). We also summarized success rate and complications of open repair in the treatment of UES (Table 2).

**Table 2:** A summary of success and complication rate in open repair of UES.

**References**	**Design**	**No. of patients**	**Success rate**	**Complication rate (≥3)**	**Follow-up (median)**
van Son et al. (18)	Retrospective	26	64.3%	12%	34
Ahmed et al. (13)	Retrospective	6	100%	23%	23
Schondorf (16)	Retrospective	35	91%	17.14%	29
Nassar and Alsafa (4)	Retrospective	32	78%	NA	47
Milhoua et al. (17)	Retrospective	7	71.4%	NA	21
Laven et al. (19)	Retrospective	15	80%	26.67%	35
Dimarco et al. (22)	Retrospective	27	76%	7.41%	24^*^
Vandenbroucke (29)	Retrospective	11	90.09%	NA	10.8^*^
Kramolowsky et al. (10)	Retrospective	7	85.7%	42.86%	16^*^

* *Mean follow-up; NA, not applicable*.

### Success Rate Distinguished by Stricture Length

**Figure 2:**
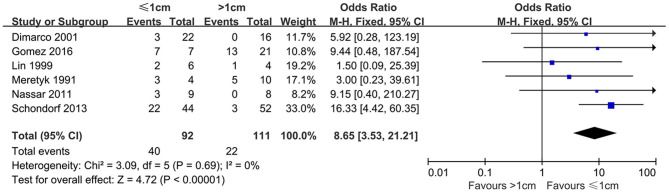
Success rate distinguished by stricture length.

Six studies reported the influence of different stricture lengths on efficacy of endoscopic produces. According to the pooled articles, the average success rate was 43.48% in patients with ≤ 1-cm strictures; however, patients with >1 cm length was 19.82%. Compared to the subgroup of patients with >1-cm strictures, the success rate was significantly higher for ≤ 1 cm ones (OR, 8.65; 95% CI, 3.53–21.21; *P* < 0.00001) (Figure 2).

### Success Rate Distinguished by Stricture Side

**Figure 3:**
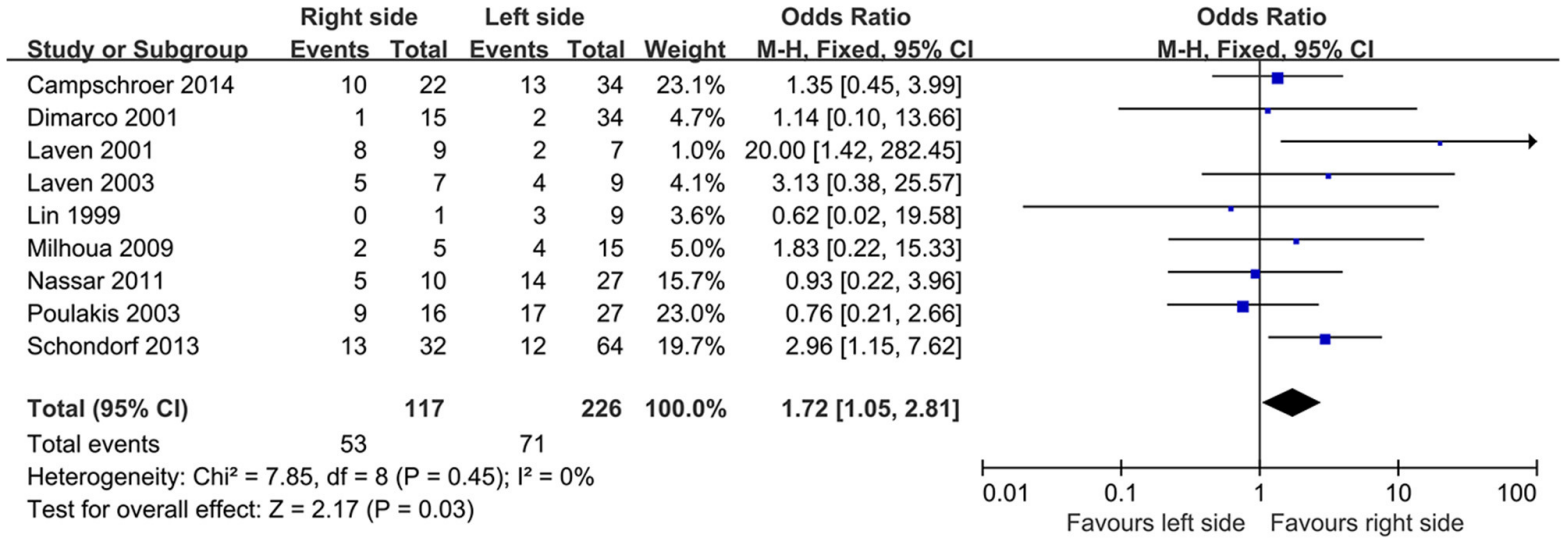
Success rate distinguished by stricture side.

Nine studies compared the efficacy of endoscopic management in different sides of UES. Based on included articles, the patients treated with endoscopic procedures in the right side had a significantly higher success rate compared with the left side (OR, 1.72; 95% CI, 1.05–2.81; *P* = 0.03) (Figure 3).

### Complications

**Figure 4:**
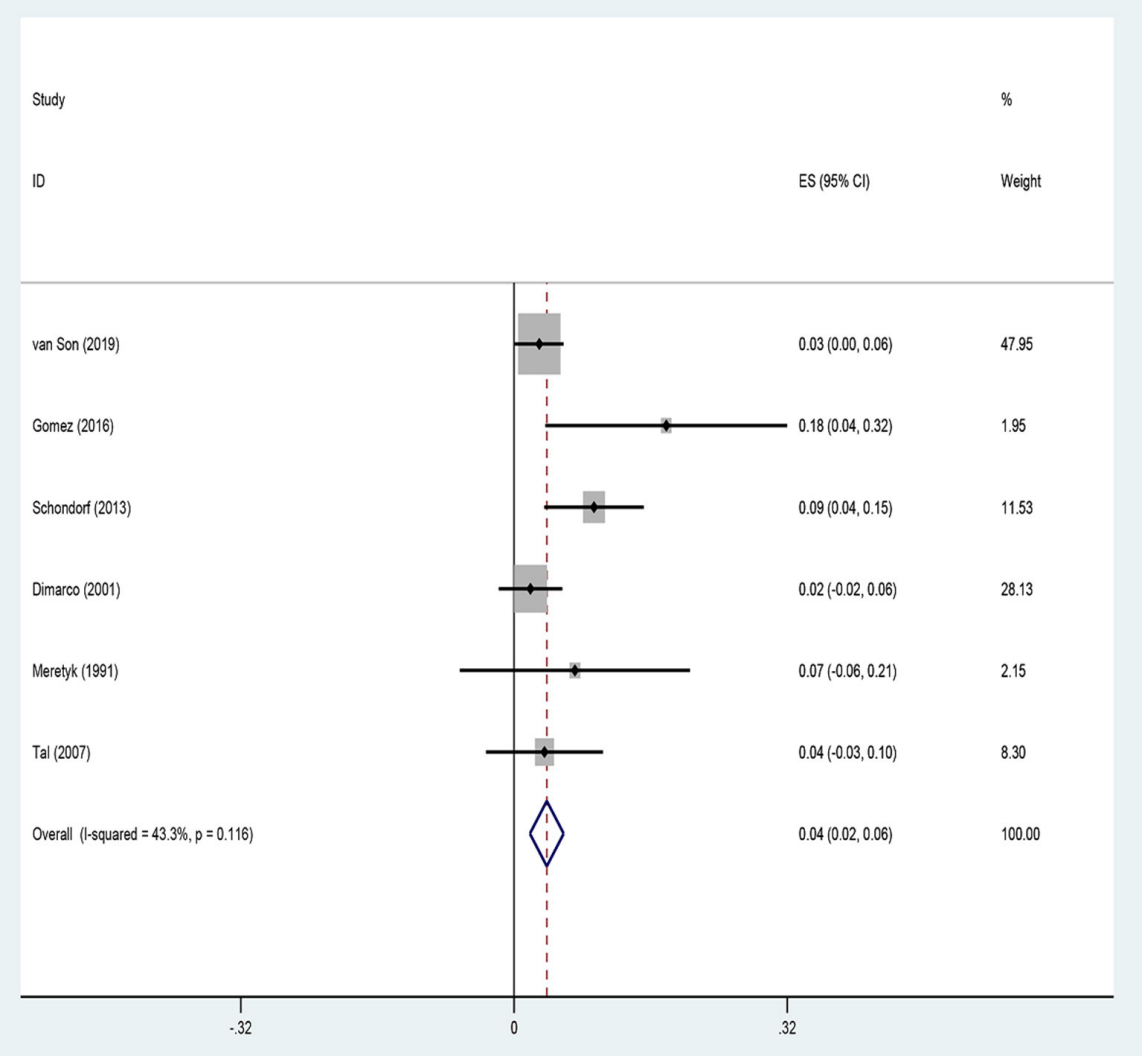
Complications of included studies.

Six studies reported the complications of endoscopic management in UES. The common Clavien–Dindo complications ≥3 were urinary tract infections, urosepsis, bleeding, and metabolic decompensation. Our meta-analysis showed a complication rate of 3.8% after endoscopic procedures, using a fixed-effects model (Figure 4).

### Publication Bias

**Figure 5:**
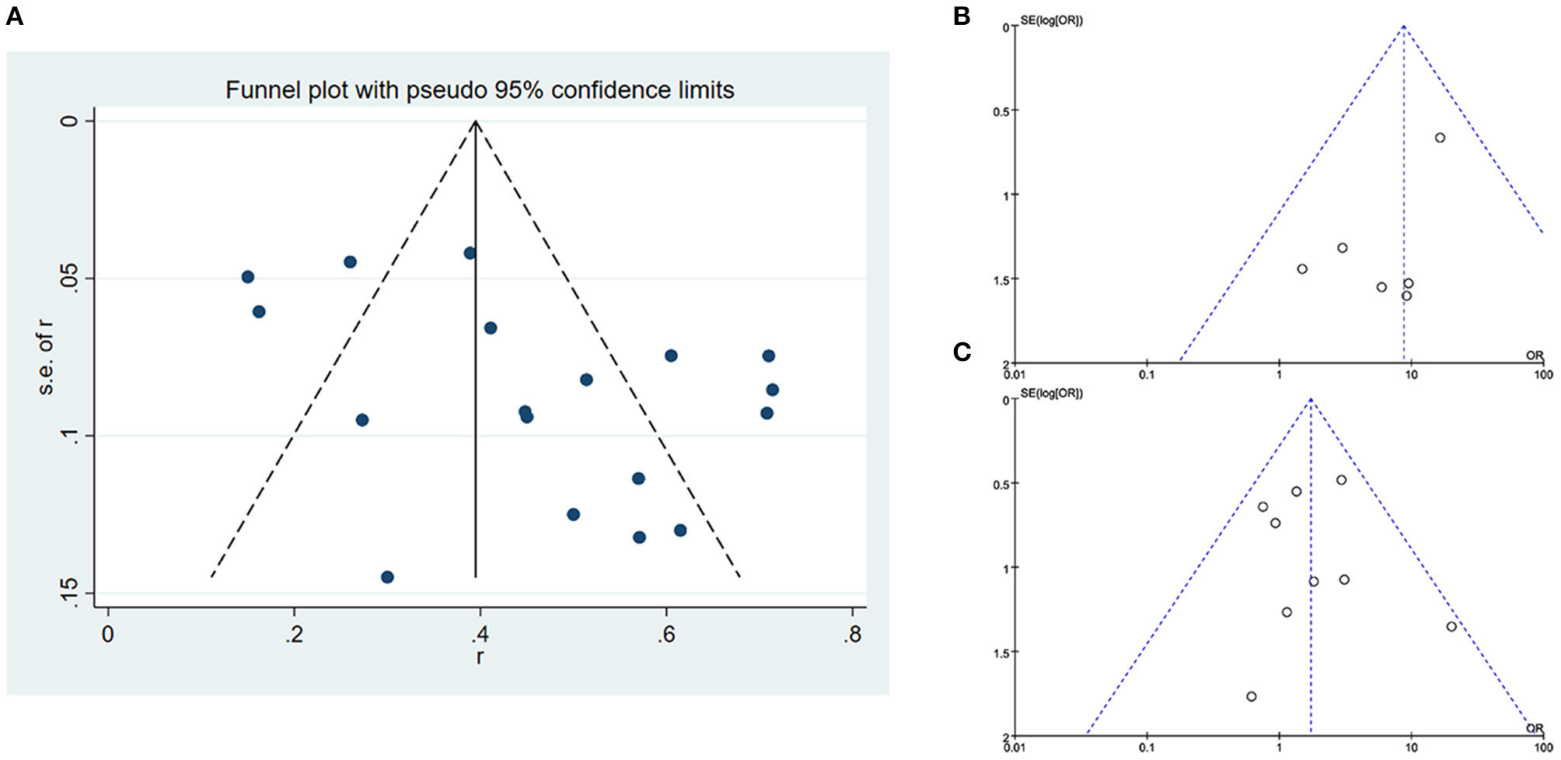
Publication bias **(A)** funnel plot of overall success rate of included studies, **(B)** funnel plot of success rate distinguished by stricture length, and **(C)** funnel plot of success rate distinguished by stricture side.

According to the funnel plots, although a publication bias exists in overall success rate. No obvious publication bias was found in our other results (Figure 5).

## Discussion

UES is a potential challenge to urologists due to its frequency, loss of renal function, and complicated infection. Factors that predict the development of UES are poorly understood, although several studies have attempted to identify such risk factors. Obesity, however, has been indicated to be associated with the formation of UES. One study demonstrated that patients with a high BMI were more likely to develop a stricture (30). Previous history of radiotherapy has also been linked to stricture formation. Knap et al. (31) reported that patients who underwent pelvic radiotherapy before had a higher risk to form a stricture than those without. This evidence has also been enhanced by Son et al. (12), who showed that the history of pelvic radiotherapy was significant of stricture recurrence in the multivariable Cox proportional hazards regression analysis.

Previously, open repair was recommended for persisting long-term patency rate. However, its inevitable complications, requirement of general anesthesia, and long hospital stay inspire us to make other alternatives. These endoscopic techniques have the advantage of reduced blood loss and short hospital stay compared with open repair. The utilization of robot-assisted surgery is increasing in urinary system diseases; therefore, we explored the efficacy of robotic reimplantation in UES. The results of a single-center retrospective analysis of Lee et al. (32) showed that the success rate of indocyanine green (ICG)-guided robotic reconstruction was 80%, and the incidence of Clavien > 2 was 25%. Among them, there were three cases of left stenosis and two cases of bilateral stenosis. The median operation time was 205 min. Although the study only included eight patients, it really provided us with a new strategy for the treatment of UES, especially for patients with left stenosis or long length stenosis.

Our meta-analysis discovered that studies published before 2000 mainly focused on evaluating the technical feasibility of endoscopic surgery. The pooled analysis showed that the overall success rate was 46%, indicating that it is technically available to treat UES using this minimally invasive procedures. Our results demonstrated that the continuous development of endoscopic devices and accumulation of surgery experience can now acquire satisfactory outcomes at reasonably high rates. Moreover, the success rate was significantly higher in patients with stricture length <1 cm than others. We speculated that chronic inflammation can be a risk factor for UES formation, which leads to the hyperplasia and fibrosis of ureter. Therefore, long length stricture is difficult to deal with endoscopic approach alone. Collectively, right side operation had a superior success rate compared with the left side. An increased incidence of left vs. right UESs has been reported by some investigators, and our results are similar to numerous studies that have reported a worse success rate for interventions on the left side. We could not rule out the heterogeneity of operative technique, because surgeons with variable experience and different methods were involved. It could also be a result of additional mobilization and tension caused by left ureter sutured with ileal conduit (3).

In addition, we reported a different success rate in our meta-analysis regarding various surgery methods. The current endoscopic standard remains incision of the stricture segment along with balloon catheter dilatation. Dilation of UES can be repeated with balloons at the exact site of the stricture and held at designated pressures under direct vision. It remains unclear which parameter can affect outcomes. Balloon size, inflation time, pressure, and number of cycles have all been reported to be associated with success rate (33–35). Several studies examining endoureterotomy with holmium laser have yielded promising outcomes. Performing under vision directly with the laser vaporization for UES has become a popular procedure with a relative high success rate, while stent insertion has a higher success rate reported by our results, especially metal stent. However, we must emphasize that this method requires ongoing interventions every 2–3 months indefinitely. Given the retrospective nature of this analysis, there is significant heterogeneity in follow-up protocols and duration of follow-up. Long-term stent exchange is potentially associated with recurrent urinary tract infection and even renal function deterioration. It could also cause some other problems such as obstruction or migration of the stent, which subjects patients to a second intervention (15). This may compromise our conclusion according to the definition of success in the treatment of UES.

From the perspective of safety of endoscopic procedures, we demonstrated that it is a relative secure approach in UES. Son et al. (12) reported 27% Clavien 2 complications in the open repair group of UES, and 8% Clavien 3b complications occurred. Nassar et al. (4) described that about 10% serious intraoperative complications happened in the open repair group. In a word, compared to endoscopic procedures, there were more complications in the open surgery group, and the complications were more severe.

However, given its moderate success rate of endoscopic treatment in UES, we need to further discuss the measures after the failure of endoscopic treatment for rescuing the renal function. Gupta et al. (36) explained a possible role that severe extrinsic fibrosis and intrinsic fibrotic stenosis cause endoscopic treatment failure. It also increased the complications of surgical reconstruction, blood loss, and operation time, leading to the deterioration of the patient's renal function. The authors also suggested that patients with high-grade hydronephrosis and poor initial renal function should avoid endoscopic treatment because of low success rate. It is meaningful for us to distinguish the potential pitfalls in the management of UES.

We admitted some limitations on our meta-analysis. Firstly, the included articles are all retrospective studies, restricting the level of evidence. Secondly, we failed to evaluate the renal function after this less invasive surgery. It may cause some doctors to blindly pursue the surgical method without predicting the possible consequences. Finally, the articles reported that the follow-up time is relatively short, and it is doubtful whether it can maintain long-term patency. All the limitations above could compromise the value of our meta-analysis.

## Conclusion

Our pooled studies showed that endoscopic procedure had an average success rate of 46% in the treatment of UES. It also indicated that endoscopic operation is feasible and associated with a moderate success rate along with a relatively low incidence of perioperative complications in the treatment of UES, especially with length ≤ 1 cm and right side, although there is still no consensus on the endoscopic technique for UES regarding balloon dilatation and laser vaporization.

## Data Availability Statement

The original contributions presented in the study are included in the article/Supplementary Material, further inquiries can be directed to the corresponding author/s.

## Author Contributions

XL, YW, and MC conceived and designed the experiments. XL, YW, QC, DX, and HZ participated in the experiments and drafted the manuscript. XL, YW, and DX contributed to the interpretation of the data. QC and HZ performed the statistical analysis. YW and MC revised the manuscript. All authors read and approved the final manuscript.

## Conflict of Interest

The authors declare that the research was conducted in the absence of any commercial or financial relationships that could be construed as a potential conflict of interest.

## References

[1] Witjes JA Bruins HM Cathomas R Compérat EM Cowan NC Gakis G. European association of urology guidelines on muscle-invasive and metastatic bladder cancer: summary of the 2020 guidelines. Eur Urol. (2020) 79:82–104. 10.1016/j.eururo.2020.03.055 32360052

[2] Anderson CB Morgan TM Kappa S Moore D Clark PE Davis R. Ureteroenteric anastomotic strictures after radical cystectomy-does operative approach matter? J Urol. (2013) 189:541–7. 10.1016/j.juro.2012.09.034 23260561

[3] Richards KA Cohn JA Large MC Bales GT Smith ND Steinberg GD. The effect of length of ureteral resection on benign ureterointestinal stricture rate in ileal conduit or ileal neobladder urinary diversion following radical cystectomy. Urol Oncol. (2015) 33:65.e1–8. 10.1016/j.urolonc.2014.05.015 25023788

[4] Nassar OA Alsafa ME. Experience with ureteroenteric strictures after radical cystectomy and diversion: open surgical revision. Urology. (2011) 78:459–65. 10.1016/j.urology.2011.01.040 21492915

[5] Shimko MS Tollefson MK Umbreit EC Farmer SA Blute ML Frank I. Long-term complications of conduit urinary diversion. J Urol. (2011) 185:562–7. 10.1016/j.juro.2010.09.096 21168867

[6] Madersbacher S Schmidt J Eberle JM Thoeny HC Burkhard F Hochreiter W. Long-term outcome of ileal conduit diversion. J Urol. (2003) 169:985–90. 10.1097/01.ju.0000051462.45388.14 12576827

[7] Kouba E Sands M Lentz A Wallen E Pruthi RS. A comparison of the Bricker versus Wallace ureteroileal anastomosis in patients undergoing urinary diversion for bladder cancer. J Urol. (2007) 178 (3 Pt 1):945–8. Discussion 948–9. 10.1016/j.juro.2007.05.030 17632159

[8] Katkoori D Samavedi S Adiyat KT Soloway MS Manoharan M. Is the incidence of uretero-intestinal anastomotic stricture increased in patients undergoing radical cystectomy with previous pelvic radiation? BJU Int. (2010) 105:795–8. 10.1111/j.1464-410X.2009.08835.x 19725823

[9] Osman Y Abol-Enein H El-Mekresh M Gad H Elhefnawy A Ghoneim M. Comparison between a serous-lined extramural tunnel and T-limb ileal procedure as an antireflux technique in orthotopic ileal substitutes: a prospective randomized trial. BJU Int. (2009) 104:1518–21. 10.1111/j.1464-410X.2009.08574.x 19388994

[10] Kramolowsky EV Clayman RV Weyman PJ. Management of ureterointestinal anastomotic strictures: comparison of open surgical and endourological repair. J Urol. (1988) 139:1195–8. 10.1016/S0022-5347(17)42857-6 3373585

[11] Slim K Nini E Forestier D Kwiatkowski F Panis Y Chipponi J. Methodological index for non-randomized studies (minors): development and validation of a new instrument. ANZ J Surg. (2003) 73:712–6. 10.1046/j.1445-2197.2003.02748.x 12956787

[12] van Son MJ Lock MT Peters M van de Putte EF Meijer RP. Treating benign ureteroenteric strictures: 27-year experience comparing endourological techniques with open surgical approach. World J Urol. (2019) 37:1217–23. 10.1007/s00345-018-2475-4 30232554 PMC6533231

[13] Ahmed YE Hussein AA May PR Ahmad B Ali T Durrani A. Natural history, predictors and management of ureteroenteric strictures after robot assisted radical cystectomy. J Urol. (2017) 198:567–74. 10.1016/j.juro.2017.02.3339 28257782

[14] Gomez FD Thomas A Sempels M Nechifor V Hubert C Leruth J. Outcomes following first-line endourologic management of ureteroenteric anastomotic strictures after urinary diversion: a single-center study. Urology. (2017) 102:38–42. 10.1016/j.urology.2016.10.009 27765587

[15] Campschroer T M.Lock TWT Lo RTH Bosch JLHR. The Wallstent: long-term follow-up of metal stent placement for the treatment of benign ureteroileal anastomotic strictures after Bricker urinary diversion. BJU Int. (2014) 114:910–5. 10.1111/bju.12729 24602310

[16] Schöndorf D Meierhans-Ruf S Kiss B Giannarini G Thalmann GN Studer UE. Ureteroileal strictures after urinary diversion with an ileal segment—is there a place for endourological treatment at all? J Urol. (2013) 190:585–90. 10.1016/j.juro.2013.02.039 23454401

[17] Milhoua PM Miller NL Cookson MS Chang SS Smith JA Herrell SD. Primary endoscopic management versus open revision of ureteroenteric anastomotic strictures after urinary diversion–single institution contemporary series. J Endourol. (2009) 23:551–5. 10.1089/end.2008.0230 19193136

[18] Tal R Sivan B Kedar D Baniel J. Management of benign ureteral strictures following radical cystectomy and urinary diversion for bladder cancer. J Urol. (2007) 178:538–42. 10.1016/j.juro.2007.03.142 17570422

[19] Laven BA O Connor RC Gerber GS Steinberg GD. Long-term results of endoureterotomy and open surgical revision for the management of ureteroenteric strictures after urinary diversion. J Urol. (2003) 170 (4 Part 1):1226–30. 10.1097/01.ju.0000086701.68756.8f 14501730

[20] Poulakis V Witzsch U De Vries R Becht E. Cold-knife endoureterotomy for nonmalignant ureterointestinal anastomotic strictures. Urology. (2003) 61:512–7. 10.1016/S0090-4295(02)02503-7 12639634

[21] Watterson JD Sofer M Wollin TA Nott L Denstedt JD. Holmium: YAG laser endoureterotomy for ureterointestinal strictures. J Urol. (2002) 167:1692–5. 10.1097/00005392-200204000-00025 11912389

[22] DiMarco DS LeRoy AJ Thieling S Bergstralh EJ Segura JW. Long-term results of treatment for ureteroenteric strictures. Urology. (2001) 58:909–13. 10.1016/S0090-4295(01)01420-0 11744456

[23] Laven BA O'Connor RC Steinberg GD Gerber GS. Long-term results of antegrade endoureterotomy using the holmium laser in patients with ureterointestinal strictures. Urology. (2001) 58:924–9. 10.1016/S0090-4295(01)01396-6 11744460

[24] Lin DW Bush WH Mayo ME. Endourological treatment of ureteroenteric strictures: efficacy of Acucise endoureterotomy. J Urol. (1999) 162 (3 Pt 1):696–8. 10.1097/00005392-199909010-00017 10458345

[25] Ravery V de la Taille A Hoffmann P Moulinier F Hermieu JF Delmas V. Balloon catheter dilatation in the treatment of ureteral and ureteroenteric stricture. J Endourol. (1998) 12:335–40. 10.1089/end.1998.12.335 9726399

[26] Cornud F Lefebvre JF Chretien Y Helenon O Casanova JM Moreau JF. Percutaneous transrenal electro-incision of ureterointestinal anastomotic strictures: long-term results and comparison of fluoroscopic and endoscopic guidance. J Urol. (1996) 155:1575–8. 10.1016/S0022-5347(01)66130-5 8627826

[27] Meretyk S Clayman RV Kavoussi LR Kramolowsky EV Picus DD. Endourological treatment of ureteroenteric anastomotic strictures: long-term followup. J Urol. (1991) 145:723–7. 10.1016/S0022-5347(17)38435-5 2005687

[28] Shapiro MJ Banner MP Amendola MA Gordon RL Pollack HM Wein AJ. Balloon catheter dilation of ureteroenteric strictures: long-term results. Radiology. (1988) 168:385–7. 10.1148/radiology.168.2.3393656 3393656

[29] Vandenbroucke F Van Poppel H Vandeursen H Oyen R Baert L. Surgical versus endoscopic treatment of non-malignant uretero-ileal anastomotic strictures. Br J Urol. (1993) 71:408–12. 10.1111/j.1464-410x.1993.tb15982.x 8499983

[30] Yang DY Boorjian SA Westerman MB Tarrell RF Thapa P Viers BR. Persistent, long-term risk for ureteroenteric anastomotic stricture formation: the case for long term follow-up. Transl Androl Urol. (2020) 9:142–0. 10.21037/tau.2019.09.05 32055478 PMC6995927

[31] Knap MM Lundbeck F Overgaard J. Early and late treatment-related morbidity following radical cystectomy. Scand J Urol Nephrol. (2004) 38:153–60. 10.1080/00365590310020060 15204405

[32] Lee Z Sterling ME Keehn AY Lee M Metro MJ Eun DD. The use of indocyanine green during robotic ureteroenteric reimplantation for the management of benign anastomotic strictures. World J Urol. (2019) 37:1211–6. 10.1007/s00345-018-2493-2 30229414

[33] Richter F Irwin RJ Watson RA Lang EK. Endourologic management of benign ureteral strictures with and without compromised vascular supply. Urology. (2000) 55:652–7. 10.1016/S0090-4295(00)00484-2 10792072

[34] Corcoran AT Smaldone MC Ricchiuti DD Averch TD. Management of benign ureteral strictures in the endoscopic era. J Endourol. (2009) 23:1909–12. 10.1089/end.2008.0453 19811059

[35] Wolf JJ Elashry OM Clayman RV. Long-term results of endoureterotomy for benign ureteral and ureteroenteric strictures. J Urol. (1997) 158 (3 Pt 1):759–64. 10.1097/00005392-199709000-00018 9258075

[36] Gupta M Tuncay OL Smith AD. Open surgical exploration after failed endopyelotomy: a 12-year perspective. J Urol. (1997) 157:1613–8. Discussion 1618–9. 10.1016/S0022-5347(01)64808-0 9112488

