# Supplementary material for: Endoscopic Procedures in the Treatment of Ureteroenteric Anastomotic Strictures: A Systematic Review and Meta-Analysis
Source: Front Surg. 2021 Apr 14;8:626939. doi: 10.3389/fsurg.2021.626939 (PMC8079934; doi:10.3389/fsurg.2021.626939)
Supplement: Supplementary file 1 [file Table_1.doc]

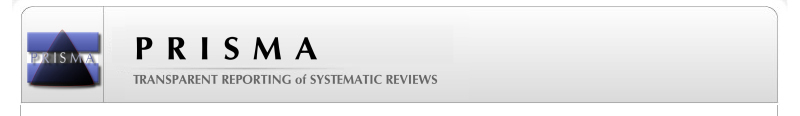
**PRISMA 2009 Flow Diagram**

**Screening**

**Included**

**Eligibility**

**Identification**

Records identified through database searching
(n = 125 )

Additional records identified through other sources
(n = 3 )

Records after duplicates removed
(n = 73 )

Records screened
(n = 73 )

Records excluded
(n = 52 )

Full-text articles assessed for eligibility
(n = 21 )

Full-text articles excluded, with reasons
(n = 3 )

Studies included in qualitative synthesis
(n = 18 )

Studies included in quantitative synthesis (meta-analysis)
(n = 18 )
